# Comparisons of serum miRNA expression profiles in patients with diabetic retinopathy and type 2 diabetes mellitus

**DOI:** 10.6061/clinics/2017(02)08

**Published:** 2017-02

**Authors:** Jianping Ma, Jufang Wang, Yanfen Liu, Changyi Wang, Donghui Duan, Nanjia Lu, Kaiyue Wang, Lu Zhang, Kaibo Gu, Sihan Chen, Tao Zhang, Dingyun You, Liyuan Han

**Affiliations:** I Shenzhen Nanshan Center for Chronic Disease Control, Department of Chronic Disease Prevention and Control, Shenzhen, 518054, China; II Ningbo Medical Center Lihuili Eastern Hospital, Department of Endocrinology, Ningbo 315040, China; III Ningbo University, School of Medicine, Zhejiang Provincial Key Laboratory of Pathophysiology, Ningbo 315211, China; IV Kunming Medical University, Department of Science and Technology, Kunming, 650500, China

**Keywords:** Type 2 diabetes mellitus, Diabetic retinopathy, Serum miRNAs

## Abstract

**OBJECTIVES:**

The aim of this study was to compare the expression levels of serum miRNAs in diabetic retinopathy and type 2 diabetes mellitus.

**METHODS:**

Serum miRNA expression profiles from diabetic retinopathy cases (type 2 diabetes mellitus patients with diabetic retinopathy) and type 2 diabetes mellitus controls (type 2 diabetes mellitus patients without diabetic retinopathy) were examined by miRNA-specific microarray analysis. Quantitative real-time polymerase chain reaction was used to validate the significantly differentially expressed serum miRNAs from the microarray analysis of 45 diabetic retinopathy cases and 45 age-, sex-, body mass index- and duration-of-diabetes-matched type 2 diabetes mellitus controls. The relative changes in serum miRNA expression levels were analyzed using the 2^-ΔΔCt^ method.

**RESULTS:**

A total of 5 diabetic retinopathy cases and 5 type 2 diabetes mellitus controls were included in the miRNA-specific microarray analysis. The serum levels of miR-3939 and miR-1910-3p differed significantly between the two groups in the screening stage; however, quantitative real-time polymerase chain reaction did not reveal significant differences in miRNA expression for 45 diabetic retinopathy cases and their matched type 2 diabetes mellitus controls.

**CONCLUSION:**

Our findings indicate that miR-3939 and miR-1910-3p may not play important roles in the development of diabetic retinopathy; however, studies with a larger sample size are needed to confirm our findings.

## INTRODUCTION

Diabetic retinopathy (DR) is a common microvascular complication of diabetes and the leading cause of legal blindness among people of working age in the Western world 1. Due to the increased lifespan of diabetic patients, the prevalence of DR will continue to increase as a result of the longer duration of diabetes. However, the molecular mechanisms underlying DR are not clearly understood. It has been reported that 28.8% of diabetic patients develop DR, whereas 22.2% of individuals with a history of diabetes do not develop DR regardless of glycemic exposure, indicating that genetic factors may play a role in the development of DR 2.

MicroRNAs (miRNAs) are a class of highly conserved, endogenous RNA sequences that regulate the activity of target mRNAs and control gene expression at the post-transcriptional level 3. Dysregulated miRNA expression has been identified as a risk factor for hepatocellular carcinoma and ischemic stroke 4,5, and studies have shown that miRNAs play a significant role in the development of diabetes and its associated complications 6. For example, circulating levels of miR-126-3p were found to be lower in patients with type 2 diabetes mellitus (T2DM) than in healthy controls 7, while circulating miR-146a levels were significantly elevated in newly diagnosed T2DM patients compared with healthy controls 8.

To date, few studies have investigated the relationship between circulating miRNA levels and the development of DR 9, with most studies focusing on diabetic rat models or endothelial cells cultured in high-glucose conditions 10. Circulating miRNA levels can be used for the early prediction of DR with high sensitivity and specificity 11, and aberrant circulating miRNA levels may represent a novel non-invasive biomarker for the early detection of DR 12. Therefore, the aim of this study was to compare the circulating miRNA profiles of DR cases and matched T2DM controls.

## MATERIALS AND METHODS

### Study subjects

The subjects were identified by community health service centers (CHSCs) under supervision of the Shenzhen Nanshan Center for Chronic Disease Control in the Nanshan district. All of the subjects provided informed consent, and the study was approved by the Ethical Committee of the Shenzhen Nanshan Center for Chronic Disease Control (2011001). All of the subjects underwent fundus fluorescein angiography, which was completed by certified ophthalmologists. The cases were T2DM patients with DR, and the controls were T2DM patients without DR. T2DM was diagnosed according to the 2010 guidelines from the American Diabetes Association 13. Subjects with acute or chronic inflammatory disease, type 1 diabetes, maturity-onset diabetes of the young, or mitochondrial diabetes were excluded.

Additionally, age, sex, BMI (body mass index), and family history of diabetes were recorded. All of the subjects underwent a general physical examination. Peripheral blood samples were collected following a 12-hour fast, and glycosylated hemoglobin (HbA1c), low-density lipoprotein (LDL), high-density lipoprotein (HDL), total cholesterol (TC), and triglyceride (TG) levels were estimated. TC, LDL, HDL and TG were measured using standard enzymatic methods and a HITACHI 7080 automatic biochemical analyzer. BMI was calculated as [weight (kg)/ height (m)^2^].

### miRNA microarrays

The RNA samples from the 5 DR cases and 5 T2DM controls were analyzed using a μParaflo™ MicroRNA microarray assay. Fluorescence images were collected using a laser scanner (GenePix 4000B, Molecular Devices) and digitized using Array-Pro image analysis software (Media Cybernetics, Washington, USA) (Figure 2). The data were analyzed by first subtracting the background and then normalizing the signals using a LOWESS filter (Locally Weighted Regression). The results were then filtered according to the following criteria: ① *p*-value<0.05; ② at least a two-fold (|log2|>1) difference between the samples (groups), with an ideal difference ≥4 (|log2| ≥2); and ③ two groups present in the sample and a strong hybridization signal (average value≥2000). The two identified miRNAs and their target sequences (5′ to 3′) are shown in Table 1.

### RNA extraction

The serum (400 μL) isolated from each sample was centrifuged at 6,000 g and at 4°C for 15 min prior to RNA extraction. miRNA was isolated from 45 DR and 45 matched T2DM serum samples using QIAzol Lysis Reagent (Qiagen, Hilden, Germany) as part of the miRNeasy Serum/Plasma Kit (Qiagen). Then, 3.5 μl of synthetic miRNA-39 from *Caenorhabditis elegans* (cel-microRNA-39) was added to the extracted miRNA as a spike-in control (1.6 x 10^8^ copies/μl working solution) before the samples were reverse transcribed to complementary DNA. RNA concentration and purity were determined using an Agilent 2100 Bioanalyzer and RNA 6000 Nano/Pico LabChip (Agilent Technologies, Boeblingen, Germany).

### Quantitative Real-Time Polymerase Chain Reaction (qRT-PCR)

Total RNA extracted from the isolated serum was initially reverse transcribed using a miScript^®^ II RT Kit (Qiagen, Germany) according to the manufacturer’s instructions. Each reverse transcription (RT) reaction contained 1 µl of miScript Reverse Transcriptase Mix, 4 µl of 5x miScript RT Buffer, 13 µl of RNase-free water and 2 µl of RNA template. The 20 µl RT reaction was incubated at 37°C for 1 hour followed by 5 min at 95°C using an iCycler system (Bio-Rad, Hercules, CA). The cDNA was diluted 10-fold before being added to each quantitative polymerase chain reaction (qPCR), with the spiked-in cel-miR-39 serving as the external control for normalization. To improve quantification accuracy, each sample was analyzed in triplicate, and both the melting curve and amplification plot analyses were used to confirm the specificity of the reactions. Each 12.5 µl quantitative real-time PCR reaction contained 6.2 µl of SYBR Green PCR Master Mix, 1.2 µl of miScript universal primer, 1.2 µl of specific primer, 2 µl of cDNA and 1.9 µl of RNase-free water. The amplification protocol consisted of an initial activation step at 95°C for 15 min, followed by 40 cycles of 94°C for 15 s, 55°C for 30 s, and 70°C for 30 s, and was carried out on the Mx3005P qPCR system (Stratagene, USA). The levels of circulating miR-3939 (Hs_miR-3939 miScript Primer Assay, MS00023688 Qiagen, Germany) and miR-1910-3p (Hs_miR-1910-3p miScript Primer Assay, MS00016464 Qiagen, Germany) were analyzed quantitatively using the 2^-ΔΔCt^ (cycle threshold) method after normalization to the cel-microRNA-39 control 14.

### Statistical analysis

Quantitative data are expressed as the mean±standard deviation, while threshold cycle (Ct) values were determined using the melting curve analysis to measure the expression of target miRNAs. Triplicate Ct values were averaged, and the relative expression level of each miRNA was calculated using the comparative threshold cycle (Ct) method (2^-ΔΔCt^). All of the miRNA values are expressed as the mean±SD. A paired t-test was used to evaluate differences in serum miRNA levels between the two groups. Differences were considered statistically significant at *p*<0.05. The statistical analysis was performed using SPSS Statistics Version 18 (SPSS Inc., Chicago, USA) and GraphPad Prism 5 (GraphPad Software, Inc., La Jolla, CA, USA).

## RESULTS

### Characteristics of the included subjects

The demographic characteristics of the study subjects are presented in Table 2. A total of 45 DR cases and 45 T2DM controls (matched by age, sex, BMI and duration of diabetes) were included in the validation stage. There were no significant differences in family history of diabetes or in levels of TC, HDL, LDL, TG or HbA1c between the two groups.

### Comprehensive miRNA profiling and qRT-PCR validation

**Figure 1:**
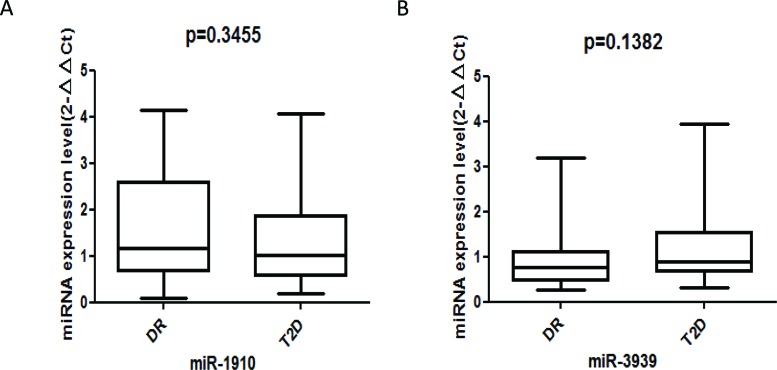
A-B: Comparison of miRNA expression levels (2^-ΔΔCt^) in the serum of DR patients (n=45) and controls (n=45). Expression levels of selected miRNAs were analyzed by qRT-PCR.

**Figure 2:**
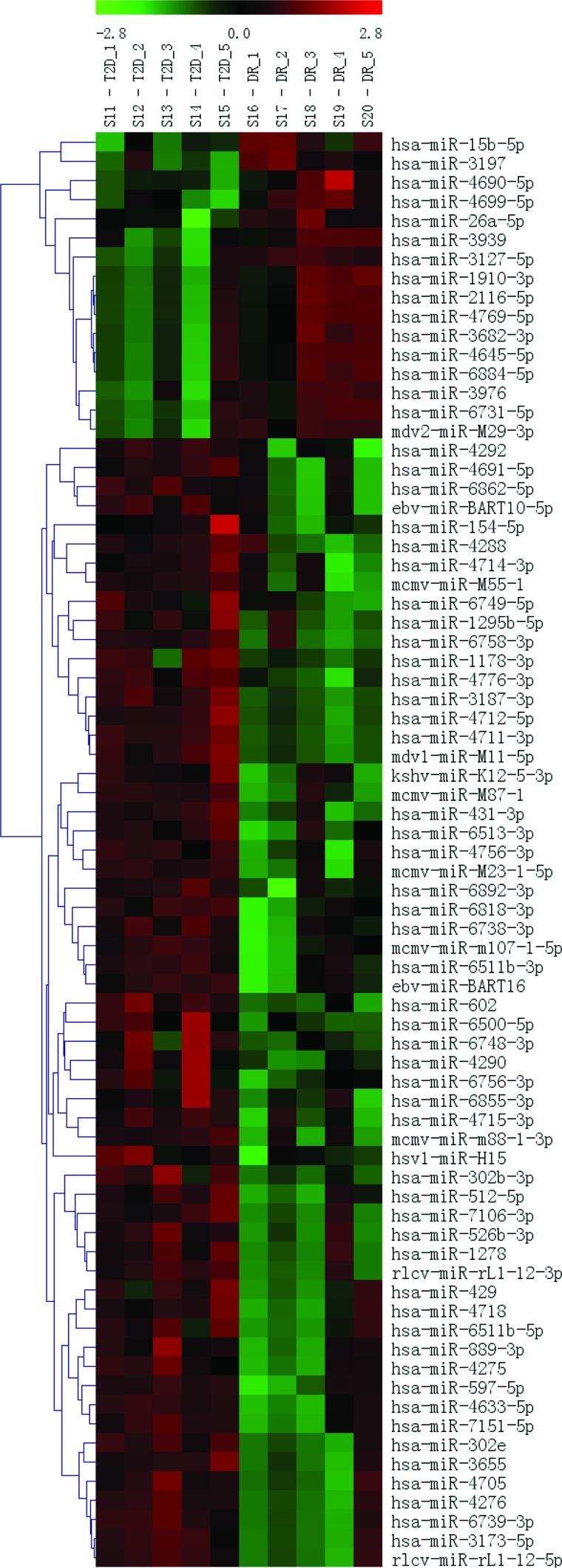
Chip cluster analysis for analyzing the differential expression of miRNAs between DR cases and T2DM controls.

To identify a DR-specific serum miRNA expression profile, the μParaflo™ MicroRNA microarray assay was used to screen for miRNAs that were differentially expressed in 5 DR cases and 5 T2DM controls. Two miRNAs (miR-3939 and miR-1910-3p) were higher in DR patients than in T2DM patients, with |log2| values of 8.58 and 8.59, respectively (Table 1). We further validated these 2 serum miRNAs in 45 DR cases and 45 matched T2DM controls using RT-qPCR; however, no statistically significant difference was found (Figure 1A-B).

## DISCUSSION

Although miR-3939 and miR-1910-3p appeared to be differentially expressed in the screening stage, qRT-PCR did not confirm these results. Consistent with our findings, Zampetaki et al. did not find a significant association between plasma miR-146a levels and T2DM 15. However, a double-blind, parallel design, placebo-controlled randomized clinical trial found that serum miR-27b and miR-320a levels were independently associated with DR susceptibility in patients with type 1 diabetes 16. Additionally, Pescador et al. 17 identified serum miR-15b, miR-138 and miR-376a as potential predictive biomarkers for obesity and T2DM. Moreover, Zhang et al. 18 showed that plasma miR-126 levels were a potential biomarker for the early prediction of T2DM susceptibility. These contradictory results may be due to differences in study design, sample collection, sample size, participant ethnicities and detection methods.

Importantly, the biological role of miRNAs in the development of DR should be noted. McArthur et al. 19 found that miR-200b regulates vascular endothelial growth factor (VEGF)-mediated abnormalities in cultured ECs and streptozotocin (STZ)-induced diabetic rats. Zhuang et al. 20 revealed that the downregulation of miR-155 attenuates retinal neovascularization via the phosphatidylinositol 3-kinase (PI3K)/Akt pathway, while Chen et al. 21 suggested that miR-410 inhibits oxygen-induced retinal neovascularization by suppressing VEGF expression. Additionally, Xiong et al. 22 determined that 17 miRNAs were dysregulated in the retinas of diabetic Sprague–Dawley rats, suggesting that miRNAs play a significant role in the progression of DR. Moreover, in high-glucose conditions, miR-152 represses VEGF and TGFβ1 expression in human retinal endothelial cells through post-transcriptional inhibition of the (pro)renin receptor 23. These studies suggest that miRNAs play a substantial role in the pathogenesis of DR.

Kong et al. reported that 7 serum miRNAs (miR-9, miR-29a, miR-30d, miR-34a, miR-124a, miR-146a and miR-375) were elevated in T2DM subjects compared to healthy controls 24, while Qing et al. 25 revealed that serum miR-21, miR-181c and miR-1179 levels could be sensitive and cost-effective biomarkers for the early detection of proliferative DR (PDR). Thus, further investigation into the circulating levels of miRNAs in samples obtained from patients with different stage disease is warranted 10.

Interestingly, Liu et al. 26 identified serum miR-126 as a biomarker for pre-diabetes and T2DM and found that six months of treatment (diet control and exercise in subjects with prediabetes or insulin plus diet control and exercise in T2DM patients) significantly increased miR-126 levels, indicating that therapeutic treatments have a significant effect on circulating miRNA levels. However, the plasma levels of 13 miRNAs (miR-15a, miR-20b, miR-21, miR-24, miR-126, miR-191, miR-197, miR-223, miR-28-3p, miR-150, miR-29b, miR-320 and miR-486) in T2DM subjects were similar before and after drug treatment (mainly sulfonylureas) 15. Further studies exploring the effects of therapeutic treatments on circulating miRNA expression levels are recommended. One limitation of the present study was the relatively small sample size. Another limitation was that the use of anti-diabetic medications was not analyzed, and pharmacological treatments may influence the expression of circulating miRNAs.

In conclusion, although the serum levels of miR-3939 and miR-1910-3p differed significantly between DR cases and T2DM controls in the screening stage, these results were not validated in the validation stage. Therefore, the above two circulating miRNAs may not play important roles in the development of DR. Further research is required to determine whether the analysis of circulating miRNA levels holds predictive value for the early detection of DR, and prospective studies investigating the biological mechanisms and effects of different therapeutic treatments should be encouraged.

## AUTHOR CONTRIBUTIONS

You D and Han L conceived and designed the study and had full access to all of the data in the study and take responsibility for the integrity of the data and the accuracy of the data analysis. Ma J, Wang J, Liu Y, Wang C, Duan D and Lu N were responsible for the data acquisition. Wang K, Zhang L, Gu K, Chen S and Zhang T were responsible for the data analysis and interpretation. Ma J, Wang C, Wang J and Liu Y were responsible for the manuscript drafting. You D and Han L were responsible for the critical revision of the manuscript for important intellectual content. Ma J, Wang C, Wang J and Liu Y were responsible for the statistical analysis. You D and Han L supervised the study.

## Figures and Tables

**Table 1:** Differential expression of serum miRNAs based on the miRNA expression microarray.

	T2DM		DR				
Assay	Mean	SD	Mean	SD	*p*-value	|log2|	Target Sequence (5′ to 3′)
hsa-miR-3939	2	3	876	812	0.02	8.58	UACGCGCAGACCA CAGGAUGUC
hsa-miR-1910-3p	3	6	697	788	0.03	8.09	GAGGCAGAAGC AGGAUGACA

SD: standard deviation; log2: the difference between the groups (DR/T2DM), which should be larger than 2; Target Sequence: the miRNA sequence.

**Table 2:** Clinical characteristics of the included DR cases and T2DM controls.

	DR (n=45)	T2D (n=45)	*p*
Age (years)	66.24±8.40	65.42±7.96	0.606
BMI (kg/m^2^)	24.48±3.71	23.9±3.12	0.293
Duration of diabetes	16.78±7.42	15.96±9.50	0.638
TC (mmol/L)	4.50±0.84	4.63±1.81	0.52
HDL (mmol/L)	1.22±0.39	1.15±0.31	0.347
LDL (mmol/L)	2.97±0.96	2.64±0.67	0.54
TG (mmol/L)	1.50±1.23	1.43±1.05	0.925
HbA1c	8.63±2.3	7.62±1.68	0.157
